# A Yeast Chemical Genetic Screen Identifies Inhibitors of Human Telomerase

**DOI:** 10.1016/j.chembiol.2012.12.008

**Published:** 2013-03-21

**Authors:** Lai Hong Wong, Asier Unciti-Broceta, Michaela Spitzer, Rachel White, Mike Tyers, Lea Harrington

**Affiliations:** 1 Wellcome Trust Centre for Cell Biology, King’s Buildings, University of Edinburgh, Mayfield Road, Edinburgh, EH9 3JR, UK; 2 Edinburgh Cancer Research UK Centre, Medical Research Council Institute of Genetics and Molecular Medicine, University of Edinburgh, Crewe Road South, Edinburgh, EH4 2XR, UK; 3 Faculty of Medicine, University of Montreal, Institute for Research in Immunology and Cancer, Chemin de Polytechnique, Montreal, Quebec, H3T 1J4 Canada

## Abstract

Telomerase comprises a reverse transcriptase and an internal RNA template that maintains telomeres in many eukaryotes, and it is a well-validated cancer target. However, there is a dearth of small molecules with efficacy against human telomerase in vivo. We developed a surrogate yeast high-throughput assay to identify human telomerase inhibitors. The reversibility of growth arrest induced by active human telomerase was assessed against a library of 678 compounds preselected for bioactivity in *S. cerevisiae*. Four of eight compounds identified reproducibly restored growth to strains expressing active human telomerase, and three of these four compounds also specifically inhibited purified human telomerase in vitro. These compounds represent probes for human telomerase function, and potential entry points for development of lead compounds against telomerase-positive cancers.

## Graphical abstract

**Graphical Abstract:**

**Figure:**
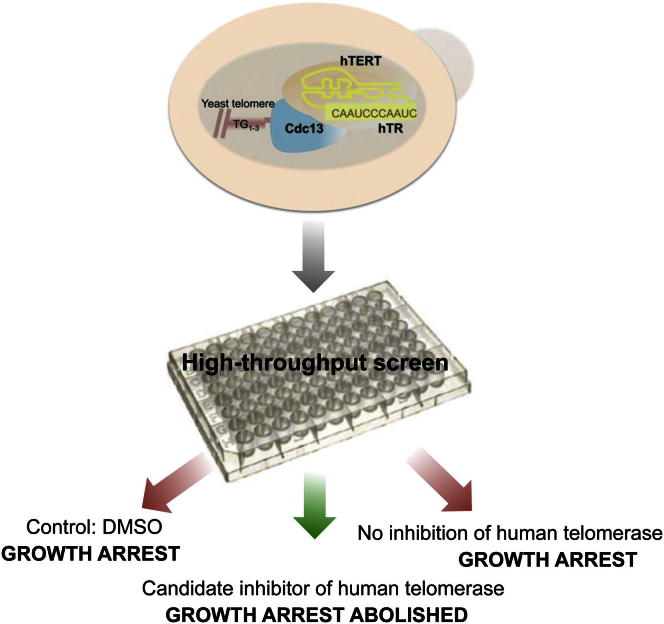

**Highlights:**

► Growth arrest induced by human telomerase in yeast is chemically reversible ► Readout is sensitive to telomerase catalytic activity and telomere recruitment ► Three cell-permeable compounds also inhibit purified human telomerase ► Yeast can be successfully used to screen for human telomerase inhibitors

## Teaser

Ability of baker's yeast to arrest cell division reversibly in response to the expression of human telomerase was used to screen for compounds that impaired the enzyme. Wong et al. found several compounds with desired activity, establishing yeast as a model system to identify new probes for telomerase function.

## Introduction

The reactivation of telomerase is important for cancer cell survival. While primary somatic cells do not normally express telomerase and undergo progressive telomere shortening that leads subsequently to cell senescence, many malignant cell types activate telomerase expression to counteract telomere attrition and support tumor growth. Given its selective expression in cancer and requirement for cell immortality, telomerase in principle represents an ideal therapeutic cancer target (Harley, 2008). Mounting preclinical and clinical data strongly suggest that telomerase inhibition is a viable approach to suppress cancer progression, particularly in cancer cells with shorter telomeres, low telomerase expression, and rapid turnover (Harley, 2008). Furthermore, telomerase inhibition augments the sensitivity of carcinoma cell lines to radiation therapy and cytotoxic drugs (Gomez-Millan et al., 2007; Röth et al., 2010; Ward and Autexier, 2005).

Approaches to inhibit telomerase in cancer cells include direct or indirect enzyme inhibition though gene therapy, oligonucleotides that are complementary to the RNA template, and small molecule antagonists of enzyme activity (Harley, 2008). The most potent known in vitro telomerase inhibitor, BIBR1532 is a nonnucleosidic compound that interferes with the processivity of telomere elongation; however, BIBR1532 has not been taken into clinical trials due to issues with solubility and bioavailability (Damm et al., 2001; El-Daly et al., 2005; Pascolo et al., 2002). Imetelstat (GRN163L) is an oligonucleotide template antagonist that targets the active site of human telomerase through the telomerase RNA (hTR) template region and is the only telomerase inhibitor of its class to have progressed to phase II clinical trial (Röth et al., 2010; Harley, 2008). Thus, there remains a pressing need for the development of additional candidate telomerase inhibitors for cancer treatment.

Here, we report a surrogate genetic screening assay for human telomerase activity based on conditional heterologous expression of the two core catalytic subunits of the human ribonucleoprotein (RNP), hTR and telomerase reverse transcriptase (hTERT) in budding yeast (Bachand and Autexier, 1999; Bah et al., 2004). When hTERT is tethered to the yeast telomere via fusion with a yeast telomeric DNA binding protein, Cdc13, growth arrest occurs upon coexpression with hTR (Bachand and Autexier, 1999; Bah et al., 2004). We exploited this inducible growth arrest phenotype in a high-throughput assay to identify three compounds that reverse growth suppression via the specific inhibition of human telomerase.

## Results

### Induction of Active Human Relomerase in Budding Yeast

**Figure 1:**
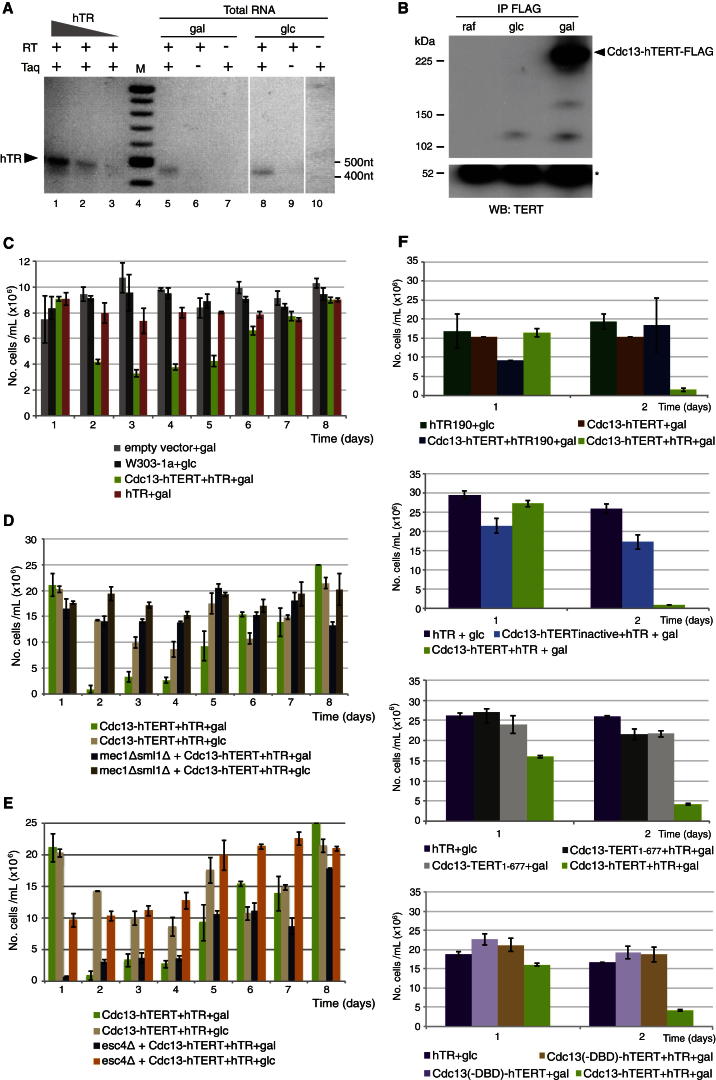
Reconstitution of Active Human Telomerase in *S. cerevisiae* via Coexpression of Wild-Type Cdc13-hTERT-FLAG and hTR (A) RT-PCR analysis of hTR expression from total cellular RNA (30 ng) prepared from a W303-1a strain containing p*CDC13-hTERT-FLAG* and p*hTR* plasmids in media containing galactose (gal; lanes 5–7) or glucose (glc; lanes 8–10), and, as a control, hTR synthesized in vitro (lanes 1–3; 0.5 ng, 0.2 ng, 0.05 ng). Irrelevant lanes between lanes 7–8 and 9–10 were omitted. RT, reverse transcriptase; Taq, Taq polymerase; M, DNA markers. (B) Immunoprecipitation (IP) of 500 μg crude lysate onto anti-FLAG resin followed by detection with anti-FLAG (Oulton and Harrington, 2004) after growth in noninducible (raffinose, raf), repressive (glc), and galactose-containing (gal) media. The predicted mass of Cdc13-hTERT-FLAG is 232 kDa, indicated by the arrow at right. Asterisk indicates immunoglobulin G heavy chain (53 kDa) of anti-FLAG antibody. (C) Cell number during an 8-day growth period of W303-1a in galactose (gal) or glucose (glc) or W303-1a in galactose containing an empty *GAL1* plasmid (empty vector), hTR alone (hTR), or Cdc13-hTERT-FLAG and hTR. Error bars indicate SD, n = 3. (D) Growth analysis as in (C) in strains expressing Cdc13-hTERT-FLAG + hTR in a *mec1*Δ *sml1*Δ strain background. Error bars indicate SD, n = 3. (E) Growth analysis as in (C) in strains expressing Cdc13-hTERT-FLAG + hTR in an *esc4*Δ strain background. Error bars indicate SD, n = 3. (F) Cell number after 2 days of growth of W303-1a strains expressing an inactive hTR mutant (hTR190), truncated, inactive hTERT (TERT_1–677_), inactive hTERT (containing an amino acid substitution in the catalytic site, D868A), or lacking the DNA-binding domain (DBD) of Cdc13. Error bars indicate SD, n = 3. See also Figure S1 and Table S1.

To reconstitute functional human telomerase in *S. cerevisiae*, we introduced p*hTR*, for constitutive expression of hTR, and p*GAL1-CDC13-hTERT-FLAG,* to permit galactose-inducible expression of *CDC13-TERT-FLAG* (Bah et al., 2004; Evans and Lundblad, 1999) (Figure S1A available online). Expression of hTR and Cdc13-hTERT-FLAG in galactose-containing medium was confirmed by reverse transcriptase (RT)-PCR and western blot (Figures 1A and 1B). Galactose induction of *CDC13-TERT-FLAG* resulted in microcolony formation and growth suppression by 48 hr, and persisted up to 96 hr (Figures 1C and S1C). No growth delay was observed during the first 24 hr, likely due to a lag in the assembly and recruitment of active human telomerase to levels sufficient to induce a response (Figure S2A; data not shown). As expected, strains expressing hTR alone, which is insufficient for human telomerase activity in yeast (Bah et al., 2004), exhibited no growth delay (Figure 1C). The growth impedance caused by human telomerase expression depended upon the presence of the ATM-like kinase Mec1, which is the predominant DNA damage checkpoint kinase in budding yeast (d’Adda di Fagagna et al., 2004) (Figures 1D and S1E). The arrest did not depend on Esc4, a key factor in DNA replication restart that is dispensable for the intra-S-phase checkpoint arrest (Rouse, 2004) (Figure 1E). Expression of human telomerase did not interfere with endogenous yeast telomerase function, since there were no changes in the terminal telomere DNA-containing restriction fragment (TRF) length and no human telomeric repeats were detected at yeast telomeres (Figure S1D; data not shown) (Bah et al., 2004). Unlike the Mec1-dependent, irreversible arrest in response to a double-strand break at a native yeast telomere (Sandell and Zakian, 1993), the growth inhibition induced by human telomerase was reversible, and growth resumed if glucose was added to the medium to suppress *CDC13-TERT-FLAG* (Figure S1H).

To confirm the specificity of the inducible growth suppression, we assessed the effect of mutations in the cDNAs encoding hTR, hTERT, and Cdc13 upon growth after confirmation that the mutant mRNA and proteins were expressed at levels comparable to their wild-type counterparts (Figures S1F and S1G). A 10-nucleotide (nt) substitution between nts 190 and 199 was introduced into hTR (hTR190), rendering it catalytically inactive (Autexier et al., 1996). Two mutations of hTERT were also introduced: the first (TERT_1–677_) lacks RT motifs essential for activity (Banik et al., 2002), and the second (hTERT-D868A-FLAG) contains an alanine substitution at D868, a residue essential for activity (Harrington et al., 1997). Finally, the telomere DNA binding domain within Cdc13, which is essential for its telomere recruitment (Evans and Lundblad, 1999), was removed from the hTERT fusion protein. All four mutations abolished the growth arrest (Figure 1F). Thus, the arrest depended on telomere recruitment and the catalytic activity of human telomerase conferred by hTR and hTERT.

### A Yeast-Based HTS for Human Telomerase Inhibitors

**Figure 2:**
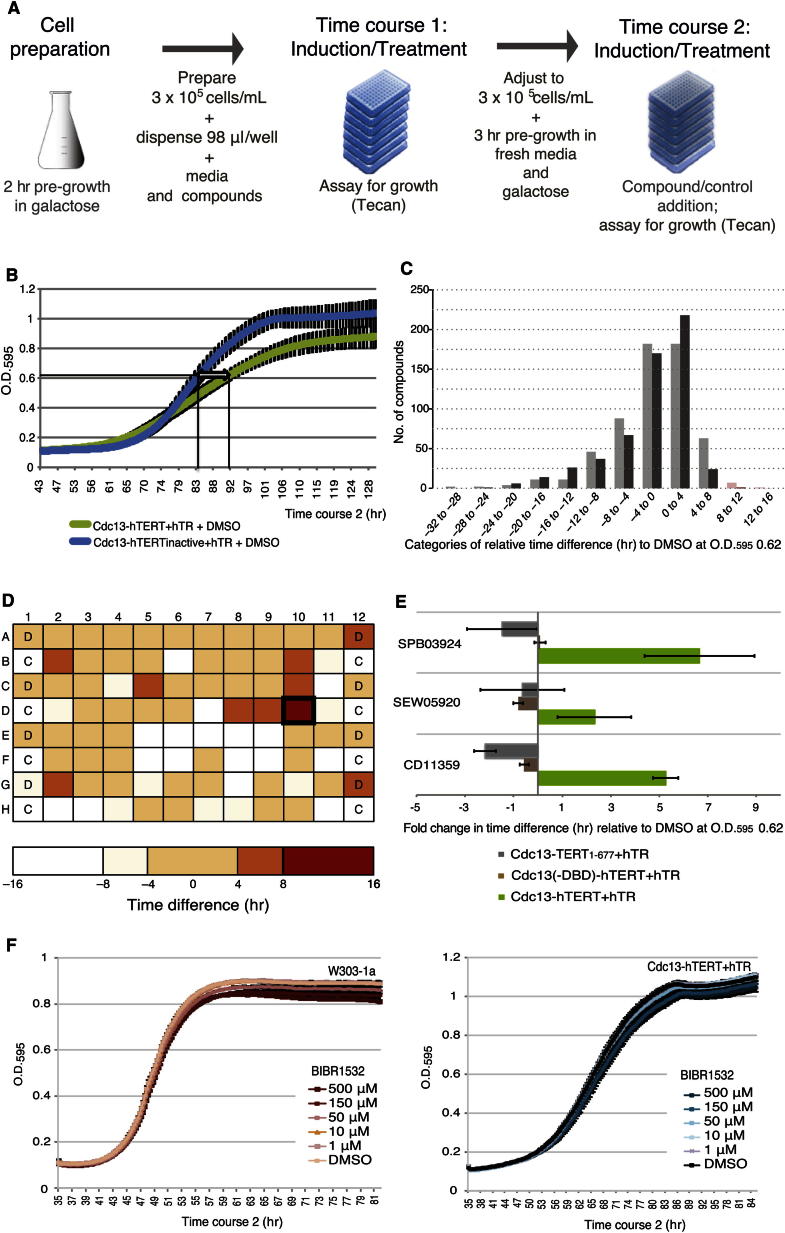
High-Throughput Chemical Screens of W303-1a Expressing Cdc13-hTERT-FLAG + hTR (A) Schematic of HTS design. Cells induced with active human telomerase were dispensed in assay plates with media containing galactose and compounds, and OD_595_ was assessed throughout two serial time courses that totaled ∼128 elapsed hr (see Experimental Procedures for details). (B) Growth profiles in a 96-well format, obtained with a Tecan shaker-reader, of W303-1a cells expressing wild-type Cdc13-hTERT-FLAG + hTR or a catalytically inactive hTERT mutant (D868A) + hTR during time course 2 (commencing at 43 hr in culture, labels spaced every 4.5 hr and rounded up or down accordingly). Horizontal double-sided arrow indicates the relative growth delay of 8.75 hr between the two strains at an OD_595_ of 0.62. Error bars, in black, indicate SD, n = 8. (C) Histogram of the number of compounds in categories of time difference (hr) to reach an OD_595_ of 0.62 relative to DMSO treatment (screen 1, light gray; screen 2, dark gray). Compounds that rescued relative growth delay by between 8 and 16 hr are shown in pink or red. (D) Heatmap analysis of time difference (hr) to reach an OD_595_ of 0.62 relative to DMSO in a representative 96-well plate during the assay phase time course 2. C, cycloheximide; D, DMSO. Wells containing compounds (or cycloheximide) that impeded growth by more than 8 hr relative to DMSO appear white. Wells in which the time to reach an OD_595_ of 0.62 relative to DMSO was advanced by 8 hr or more are red (e.g., CD11359, outlined in black). (E) Fold change in time difference (hr) to reach an OD_595_ of 0.62 relative to DMSO of strains expressing active hTERT (Cdc13-hTERT + hTR), an inactive hTERT truncation (Cdc13-TERT_1–677_ + hTR), or Cdc13 lacking its DNA binding domain [Cdc13(-DBD)-hTERT + hTR] in the presence of repurchased candidate compounds (20 μM). Values were normalized to the time rescue observed in strains expressing catalytically inactive Cdc13-hTERT(D868A)-FLAG + hTR treated with DMSO. Error bars indicate SD, n = 4. (F) Growth profiles of W303-1a cells alone (left) or expressing wild-type Cdc13-hTERT-FLAG + hTR (right) commencing at 35 hr after induction (i.e., following 32 hr of treatment during time course 1, then 3 hr in galactose only, and readdition of compound or DMSO at 35 hr) containing 500, 150, 50, 10, or 1 μM BIBR1532 or 2% v/v DMSO. Error bars indicate SD, n = 3. See also Figure S2.

**Figure 3:**
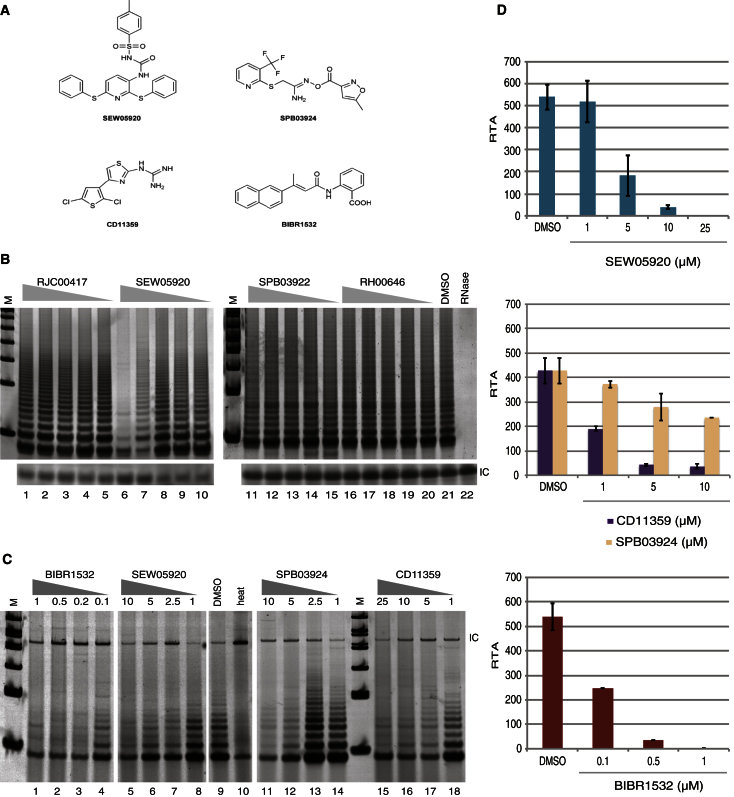
Assessment of Human Telomerase Inhibition In Vitro (A) Chemical structures of BIBR1532 and hit compounds SEW05920, SPB03924, and CD11359. (B) Representative gel of the telomerase repeat addition protocol (TRAPeze, Millipore) after addition of RJC00417, SEW05920, SPB03922, and RH00646 to recombinant human telomerase purified in vitro (Beattie et al., 1998; Oulton and Harrington, 2004) (n = 3). Lanes 1–5, treatment with 200, 100, 20, 10, or 2 μM RJC00417; lanes 6–10, treatment with 200, 100, 20, 10, or 2 μM SEW05920; lanes 11–15, treatment with 200, 100, 20, 10, or 2 μM SPB03922; lanes 16–20, treatment with 200, 100, 20, 10, or 2 μM RH00646; lane 21, treatment with 2% v/v DMSO; lane 22, pretreatment with ribonuclease A. IC; internal PCR control (36 base pairs [bp]). (C) Representative gel of the relative telomerase activity (RTA) by ELISA-TRAP (TeloTAGGG Telomerase PCR ELISA-PLUS, Roche Diagnostics) of purified human telomerase upon incubation with BIBR1532 (lanes 1–4), SEW05920 (lanes 5–8), SPB03924 (lanes 11–14), CD11359 (lanes 15–18) (concentration indicated in micromolar), 2% v/v DMSO (lane 9), or heat (lane 10). IC; internal PCR control (216 bp). (D) Levels of RTA (%) after normalization to a 216 bp internal control, as shown in (C). Error bars indicate SD, n = 3. For synthesis of BIBR1532, see Figure S2.

We used a Tecan shaker-reader platform to establish conditions in a 96-well format (Figure 2A) that recapitulated the growth delay specific to active human telomerase (compare Figure 2B and Figure 1F). Expression of hTERT and hTR was confirmed, and, as anticipated, no growth delay was observed during the first 39 hr of growth (this preassay phase is hereinafter referred to as time course 1) (Figures S2A and S2B). Following an additional 86 hr (the experimental growth assay phase, hereinafter referred to as time course 2), strains expressing catalytically active human telomerase (Cdc13-hTERT-FLAG + hTR) exhibited a delay in exponential phase growth when compared to a strain expressing inactive telomerase (hTERT-D868/A868) (Figure 2B). Using an optical density at 595 nm (OD_595_) of 0.62 as a reference for the midpoint of exponential phase (see Figure S2B), cells expressing Cdc13-hTERT-FLAG + hTR reached this OD an average of 8.75 hr later during time course 2 than strains expressing inactive hTERT [Cdc13-hTERT(D868A)-FLAG + hTR]. We next screened two independent replicates of a library of 678 bioactive compounds against the query strain expressing active human telomerase and compared growth to the same strain treated with 2% v/v dimethyl sulfoxide (DMSO). These 678 compounds were selected from a 50,000 member Maybridge library based upon a spectrum of growth inhibitory bioactivity in a drug pump-deficient yeast strain (Ishizaki et al., 2010). This bioactive library yields a hit rate of 12% in a HeLa cell proliferation assay, such that most of the compounds are nontoxic to human cells at the screening concentration used (M.S., Jan Wildenhain, Sonam Dolma, David Bellows, and M.T., unpublished data). In each 96-well assay plate, growth of the query strain in the presence of compound (20 μM), beginning at the onset of the experimental growth phase (i.e., time course 2, from 43 hr onward), was normalized against DMSO treatment (Figures 2C and 2D; Figures S2D and S2E). As expected, the growth rate of the query strain was consistent between both screens and individual assay plates upon treatment with DMSO (Figure S2D); however, the growth rates upon treatment with compounds between the two screens was more variable, with a Pearson correlation coefficient of 0.4 (Figure S2E). Relative to DMSO, a total of eight compounds rescued the time to reach an OD_595_ of 0.62 by at least 8 hr (Figure 2C), with a predicted false-positive rate of 1.4% (see Experimental Procedures). Follow-up screens that included the eight putative hits (and 43 other compounds that did not initially meet the 8-hr threshold) confirmed that three of the eight high-throughput screen (HTS) hits reproducibly and specifically advanced the growth rate of strains expressing active human telomerase (Table 1; Figure 2E; see Supplemental Experimental Procedures for details). One of the rescreened compounds, SEW05920, did not exhibit significant growth recovery in the initial HTS but exhibited a growth recovery of ∼8 hr in follow-up screens and inhibited telomerase activity in vitro (Table 1; Figures 2E and 3). In contrast, the known in vitro telomerase inhibitor, BIBR1532 (Figures 3A and S2F), had no effect upon the growth of control strains or yeast expressing human telomerase (Figure 2F). Furthermore, in a hyperpermeable strain, *pdr1*_*DBD*_*-cyc8* (Stepanov et al., 2008), BIBR1532 was toxic (Figure S2G).

### In Vitro Characterization of Inhibitors Specific to Human Telomerase

We found that three of the four compounds (SEW05920, SPB03924, and CD11359) exhibited a dose-dependent inhibition of both purified recombinant human telomerase and telomerase activity in crude HeLa cell extracts using a telomerase repeat amplification protocol (TRAP) assay (Figures 3A–3C). The fourth compound, RJC00417, did not inhibit telomerase activity in vitro and thus may inhibit human telomerase in yeast through a mechanism distinct from catalytic inhibition (Figure 3B). As a control, we observed no inhibition of the Taq polymerase used for amplification of telomerase extension products in the TRAP (data not shown). In addition, as negative controls, two other compounds that were not identified as hits (SPB03922 and RH00646) were tested and confirmed to exert no effect upon telomerase activity in vitro (Figure 3B). Using a semiquantitative assay for the detection of telomerase elongation products (ELISA-TRAP) the in vitro half maximal inhibitory concentration (IC_50_) values of the compounds fell between 1 and 6.5 μM (Figures 3C and 3D). Similar IC_50_ values were also observed against purified recombinant murine telomerase (data not shown). These values are higher than that of BIBR1532 (IC_50_ = 0.1 μM) (Damm et al., 2001; Pascolo et al., 2002) but within a concentration range typical of first hits from commercial libraries. SEW05920, SPB03924, and CD11359 are heteroaromatic small molecules that differ structurally from BIBR1532 (Figures 3A and S2F). A search of PubChem revealed that SEW05920 had not been registered in a previous bioassay; similarly, CD11359 and SPB03924 were screened in nine and 14 bioassays, respectively, against diverse targets including toll-like receptor 4, Sphingosine 1-Phosphate receptor 1, NADPH oxidase 1, phosphatase methylesterase 1, and NOTCH1 but were not identified as hits.

## Discussion

Analysis of human enzymatic activity as a direct output of a high-throughput screen in yeast has been used previously (Ceyhan et al., 2012; Griffioen et al., 2006; Perkins et al., 2001). Our results extend the utility of budding yeast as a model system to identify and validate compounds, in particular, its utility for a large multi-subunit RNP enzyme (383 kDa, including hTERT-Cdc13 and hTR). This surrogate genetic system possesses several advantages over conventional chemical screening approaches. The yeast-based assay obviates the need for large quantities of purified human telomerase in vitro and allows the facile incorporation of mutational controls. The heterologous expression system enables human telomerase to be specifically targeted, unlike human cell line-based assays, and therefore allows rapid counterselection against compounds that exhibit off-target effects on cell growth. The yeast system also selects for bioactive small molecules with well-balanced hydrophilic-lipophilic properties due to the presence of two chemically different barriers, namely, the hydrophilic polysaccharide wall and the lipophilic phospholipid membrane. Notably, the compound library used in this study was already highly enriched for yeast cell bioavailability, which allows a substantially higher hit rate that conventional library screens (Ishizaki et al., 2010).

Aside from the general features of planar, aromatic groups that typify the Maybridge compound collection, the three putative telomerase inhibitors span diverse chemical classes that do not coincide with known telomerase inhibitors (Harley, 2008). The compounds were nontoxic in isogenic human telomerase-positive or telomerase-negative cell lines with long telomeres (Sealey et al., 2010; Taboski et al., 2012) over a concentration range of 0.5–5.0 μM after 28–35 days of continuous treatment (data not shown). Although nontoxic, longer treatment periods will be required to detect telomere shortening in human cells, since significant shortening in the presence of GRN163L can take up to 3 months (Uziel et al., 2010). The chemical properties of SEW05920, SPB03924, and CD11359 appear amenable to structure-activity-relationship analysis, which will enable the design and synthesis of more potent derivatives to be tested for efficacy in telomerase-positive human cancer cell lines, and for determination of their precise mechanism of action in vitro.

## Significance

**The specificity of yeast growth suppression for active human telomerase will also enable the screening of active hTERT variants capable of drug resistance. We note that acquired drug resistance to single-drug regimes against HIV reverse transcriptase is a prevalent concern in the treatment of HIV infection (****Penazzato and Giaquinto, 2011****). The diverse compound structures recovered in the screen suggest the possibility that more than one site can be targeted on active telomerase. Combinations of inhibitors with different mechanisms of action may exhibit additive or synergistic inhibition and impede the emergence of single-point mutations in hTERT that are drug resistant. The surrogate budding yeast system thus provides a sensitive and cost-effective assay for the identification of human telomerase inhibitors and will accelerate classical hit-to-lead chemistry in parallel with testing in human cell lines and animal models.**

## Experimental Procedures

### Construction of pGAL1-CDC13-hTERT-FLAG and phTR Plasmids

Human TERT-FLAG cDNA was PCR amplified from pCR3-FLAG-hTERT-FLAG (Beattie et al., 1998) and inserted downstream of the 3′ terminal 1.2 kilobase pairs (kbp) of *CDC13* in an intermediate StrataClone vector, to which the terminator sequence of the *ADH1* gene (ADH_ter_) was subsequently inserted downstream of the fusion protein. This fragment, CDC13_SphI_-hTERT-ADH_ter_, was inserted into pGAL1-pVL1091 (Evans and Lundblad, 1999). The hTR sequence was amplified from pUC19-hTR (Beattie et al., 2000), digested with EcoRI, and inserted into pIII426 (Good and Engelke, 1994). The final constructs were verified by DNA sequencing.

### Galactose Induction and Growth Assay

W303-1a haploid cells, *MATa ade2-1 trp1-1 leu2-3*,*112 his3-11,15 ura3-1 can1-100* (Thomas and Rothstein, 1989), or a *mec1*Δ *sml1*Δ strain, *MATa ura3-52 trp1-289 leu2-3,112 bar1::LEU2* (Makovets et al., 2004; Zhao et al., 1998), were transformed with pCDC13-hTERT-FLAG and phTR plasmids (Gietz and Woods, 2002) and were propagated on synthetic dropout liquid medium lacking uracil and leucine and containing 3% (w/v) raffinose. At an OD 600 (OD_600_) of 0.3, cells were pelleted, washed, and resuspended in selective media containing 2% (w/v) galactose (to induce expression of Cdc13-hTERT-FLAG) or 2% (w/v) glucose.

### Cdc13-hTERT-FLAG and hTR Expression by RT-PCR Analysis

Total cellular RNA was isolated as described by Burke et al. (2000) and amplified using the Sensiscript RT Kit protocol (QIAGEN); hTR, forward: 5′-GGGTTGCGGAGGGTGGGCC-3′, reverse: 5′-GCATGTGTGAGCCGAGTCCTGG-3′; hTERT, forward 5′-GCGAGCTGCGGTCACCCC-3′, reverse 5′- AGCTCCTGCAGCGAGAGC-3′; Act1, forward 5′- ATGGATTCTGAGGTTGCTGCTTTGGTTA-3′, reverse 5′- TGTTCTTCTGGGGCAACTCTCAATT-3′.

### High-Throughput Chemical Screens

W303-1a cells bearing p*CDC13-hTERT-FLAG* and p*hTR* plasmids were grown in selective medium containing 3% (w/v) raffinose until an OD_600_ of 0.3, followed by a 2 hr pregrowth in medium containing 2% (w/v) galactose prior to addition of compounds. To conduct the HTS (Figure 2A), 98 μl (3 × 10^5^ cells per milliliter) was dispensed into 96-well clear flat-bottomed microplates (Corning Costar) using a Biomek FX (Beckman Coulter). A small molecule bioactive library of 678 compounds was utilized for the screen, consisting of compounds selected from the Maybridge collection (Thermo Fisher Scientific). Two microliters of each compound from the master library plate was dispensed into the assay plate at a final concentration of 20 μM, 2% (v/v) DMSO. DMSO at 2% (v/v) and 20 μM cycloheximide (Sigma) final concentration were added as controls to each assay plate, into alternate wells in columns 1 and 12 using the Span 8 module of the Biomek FX. Nine plates were analyzed, 80 compounds per plate, with 16 wells for controls. After compound addition, the plates were sealed and followed for time course 1 (up to 39 hr) on a Sunrise plate reader (Tecan) at absorbance 595 nm, maintained at 30°C and shaking at 564 rpm. After time course 1, cells in assay plates were diluted to 3 × 10^5^ cells per milliliter in fresh media containing galactose using the Biomek FX. The plates were preincubated in galactose-containing media at 30°C for 3 hr (to allow a period of recovery from the stationary phase in the absence of compound), 2 μl of each compound or control was added to plates as described earlier, returned to the plate readers, and followed for time course 2 (up to 86 hr), for a total of ∼128 elapsed hours. In follow-up experiments, we confirmed that the ability of a validated hit (SPB03924) to accelerate growth recovery did not differ significantly when the compound was added immediately at the outset of time course 2 versus its readdition after a 3 hr period in galactose only (data not shown).

### Identification of Human Telomerase Inhibitors

Growth curves were analyzed using a growth curve analysis tool implemented in R. After smoothing of growth curves, the time required for cells induced with human telomerase to reach an OD_595_ of 0.62 in the presence of each compound during time course 2 was derived using a data-based approach (Warringer and Blomberg, 2003). The time to reach an OD_595_ of 0.62 for each compound was normalized to the average DMSO value from each assay plate (to arrive at a normalized value, x, for each compound) and used to generate heatmaps as shown in Figure 2C. Z scores were calculated based on the equation: z = (x − μ)/σ, where μ is the mean time difference for all compounds in the bioactive library and σ is the SD for all compounds in the bioactive library. The rate of false-positives (1.4%) was estimated based on the number of DMSO-treated wells that were able to accelerate the time to reach an OD_595_ of 0.62 by 8 hr or more, using the following equation: 1 − (a_true-positives_/b_total_) × 100%, where a corresponds to the number of DMSO controls that exhibited a rescue in growth equal to or above 8 hr and b corresponds to the total number of DMSO controls included in the screen. Four follow-up subscreens were performed after the high-throughput screen, leading to the identification of the validated hit compounds listed in Table 1. These compounds were also analyzed for their ability to inhibit human telomerase activity in vitro (see Supplemental Experimental Procedures for details).

## Figures and Tables

**Table 1:** Screen Hits and Corresponding Z Score Values

Compounds	Time (hr) Difference at an OD_595_ of 0.62 Relative to DMSO	Z Score
CD11359	8.3	1.7
SPB03924	12	2.3
RJC00417	10	2.0
SEW05920	8.3^a^	1.7

a SEW05920 did not significantly rescue growth in the initial HTS analysis but was validated as a hit based on significant growth recovery in two follow-up subscreens and upon repurchase of the compound from the commercial supplier. Please refer to Supplemental Experimental Procedures.

## Supplemental Information

Document S1. Figures S1 and S2, Supplemental Experimental Procedures, and Table S1
