# Supplementary material for: A Yeast Chemical Genetic Screen Identifies Inhibitors of Human Telomerase
Source: Chem Biol. 2013 Mar 21;20(3):333–40. doi: 10.1016/j.chembiol.2012.12.008 (PMC3650558; doi:10.1016/j.chembiol.2012.12.008)
Supplement: Document S1. Figures S1 and S2, Supplemental Experimental Procedures, and Table S1 [file mmc1.pdf]

Chemistry & Biology, Volume *20*

Supplemental Information

A Yeast Chemical Genetic Screen Identifies Inhibitors of Human Telomerase

Lai Hong Wong, Asier Unciti-Broceta, Michaela Spitzer, Rachel White, Mike Tyers, and Lea Harrington

<u>Inventory of Supplemental Information</u>

Figure S1 (related to Figure 1) illustrates that the inducible growth arrest observed in W303-1a cells is dependent on the catalytic activity of human telomerase via the co-expression of wild-type Cdc13-hTERT-FLAG and hTR. Figure S1A shows the strategy used to exogenously induce functional human telomerase in W303-1a. Expression of Cdc13-hTERT-FLAG + hTR activates a DNA-damage response as suggested by the resultant microcolony formation (Figure S1B-S1C), which further support the Mec1p-dependent growth arrest illustrated in Figure 1D. Lack of changes at the yeast telomere upon expression of active human telomerase (Figure S1D) supports that human telomerase at the yeast telomere does not target yeast telomere function, and hence rescue of the induced growth delay by a potential human telomerase inhibitor is a feasible output of human telomerase activity. Figure S1E confirms that the wild type-like growth observed in *mec1Δ sml1Δ* cells is not due to differential levels of Cdc13-hTERT-FLAG and hTR expression. Figure S1F-G illustrates that the growth delay in W303-1a cells is a consequence of catalytically active human telomerase and that the levels of expression of *TERT* and *hTR*, and corresponding mutants, are comparable. Figure S1H confirms that the growth arrest induced by active human telomerase is reversible upon addition of glucose (which suppresses hTERT expression) suggesting that the arrest is non-lethal and therefore it should be possible to identify compounds that reverse the growth suppression.

Figure S2 (related to Figures 2 and 3) provides additional data regarding the suitability of human telomerase expression in yeast as a system to identify human telomerase inhibitors. The co-expression of Cdc13-hTERT-FLAG and hTR recapitulates a growth arrest only after "time course 2" (86 hours continuous growth) (as in Figures 1, 2); the specificity of this latent growth delay is further supported by the lack of difference in growth rates between query and control strains after the first 39 hours growth (time course 1) (Figure S2A). RT-PCR analysis of *TERT* and *hTR* (Figure S2B) was conducted to confirm that the levels of expression under 96-well growth conditions are in agreement with previous results in Figure 1A-B. In Figure S2C, growth plots are compared using y-axes with non-logarithmic (i.e. as in Figure 2B) and logarithmic scales to further confirm that the growth delay in Figure 2B occurred during the exponential growth phase. The bioactive library consists of a total of nine 96-well assay plates and Figure S2D illustrates the growth delay

induced in the query strain expressing wild-type Cdc13-hTERT-FLAG + hTR in each assay plate upon DMSO treatment. The latter control is important to normalize the effects between compounds screened. Figure S2E illustrates the diverse effect of bioactive compounds in growth acceleration relative to DMSO between two independent screens. A Pearson correlation coefficient value of 0.4 was determined between HTS repeat 1 (n1) and repeat 2 (n2). Given the long screening period and dilution steps involved, variability between screens is predicted and thus we analyzed compounds from each individual screen that had significant Z-score values. BIBR1532 synthesis, which is summarised in Figure S2F, was necessary to conduct studies comparing the uptake of BIBR1532 in yeast to other candidate compounds. An apparent lack of uptake of BIBR1532 in W303-1a cells (Figure 2F) prompted us to assess whether the compound exert a phenotype in a more permeable cell background such as *$pdr1_{DBD}$-cyc8*; the compound was cytotoxic across most concentrations, irrespective of human telomerase expression status (Figure S2G).

Table S1 (related to Figure 1).

Supplemental Data

Supplemental Figures

Figure S1, related to Figure 1. Reconstitution of active human telomerase in W303-1a yeast. (A) hTR gene is subcloned in a high copy plasmid (p*hTR*) and constitutively expressed via the *RPR1* promoter and terminator (Good and Engelke, 1994). Fusion sequence Cdc13-hTERT-FLAG is over-expressed via the *GAL1*-inducible promoter and *ADH1* terminator (Thomas and Rothstein, 1989) in the p*GAL1-CDC13-hTERT-FLAG* plasmid. (B) Brightfield images (100X) of the indicated W303-1a strains. Microbar indicates scale. (C) Microcolony formation, after 5 days incubation at 30°C of the indicated W303-1a strains. Microbar indicates scale. (D) Telomere length analysis of W303-1a cells expressing wild-type Cdc13-hTERT-FLAG + hTR in raffinose (raf), glucose (glc) or galactose (gal) at indicated passages. The terminal restriction fragment (TRF) of 1.2 kbp is indicated with an arrow. (E) RT-PCR analysis of *TERT*, *hTR* and yeast actin (*ACT*) expression in a *mec1* Δ*sml1*Δ cells co-expressing Cdc13-hTERT-FLAG and hTR in media containing glucose (glc, lanes 1-3), galactose (gal, lanes 4-6) in reactions with or without reverse transcriptase (RT) or Taq polymerase (Taq) as indicated. (F) Expression of hTERT-FLAG in W303-1a cells co-expressing Cdc13-hTERT-FLAG and hTR190, wild-type Cdc13-hTERT-FLAG and hTR or catalytically inactive hTERT (D868A) and hTR by western blot. Immunoprecipitation of 500 μg crude lysate onto anti-FLAG resin (followed by detection with anti-FLAG) after growth in glucose (glc) and *GAL1*-inducible galactose (gal) media. The predicted mass of Cdc13-hTERT-FLAG is 232 kDa, as indicated by an arrow. Asterisk indicates IgG heavy chain (53 kDa) of anti-FLAG antibody. (G) RT-PCR analysis of hTR190, *Cdc(-DBD)-hTERT* and Cdc13-$TERT_{1-677}$, lanes as in (E). Upper gel image (*TERT* expression) between lanes 5-6 was omitted. (H) Growth analysis of W303-1a cells expressing wild-type

Cdc13-hTERT-FLAG + hTR or a W303-1a cells alone after 8 days growth in galactose (gal, represented with bars) or 4 days growth in galactose and subsequently shifted to glucose (gal/glc, represented with triangles only). Error bars indicate standard deviation, n=3.

Figure S2, related to Figures 2 and 3. HTS validation of human telomerase inhibitors. (A) Growth analysis of W303-1a cells expressing wild-type Cdc13-hTERT-FLAG + hTR or a catalytically inactive hTERT mutant (D868A) + hTR after "time course 1". Error bars indicate standard deviation, n=8. Y-axis, optical density at 595 nm ($O.D._{595}$); x-axis, time elapsed in minutes. (B) RT-PCR analysis of *TERT*, *hTR* and yeast actin (*ACT*) expression from total cellular RNA (30 ng) prepared from a W303-1a strain containing wild-type Cdc13-hTERT-FLAG + hTR (lanes 1-3) or inactive Tert (Cdc13-hTERT(D868A)-FLAG), + hTR (lanes 4-6). RT, Reverse Transcriptase; Taq, Taq polymerase; M, DNA marker. (C) Growth analysis of W303-1a cells expressing wild-type Cdc13-hTERT-FLAG + hTR or a catalytically inactive hTERT mutant (D868A) + hTR as in Figure 2B using a log scale on the y-axis (right panel). Error bars, in black, indicate standard deviation, n=8. The profile from Figure 2B was reproduced (left panel) for side-by-side comparison of the two graph methods.

(D) Average time (min) to reach $O.D._{595}$ 0.62 of W303-1a cells expressing wild-type Cdc13-hTERT-FLAG + hTR in each 96-well assay plate (1-9) exposed to DMSO during "time course 2". Error bars indicate standard deviations, n=8. (E) Scatterplot of the time to reach $O.D._{595}$ 0.62 (normalized to DMSO values) (h) of HTS repeat 1 (n1, y-axis) versus HTS repeat 2 (n2, x-axis). Red lines were plotted at 0.5 h and compounds able to rescue the growth delay relative to DMSO by 0.5 h or more are shaded in blue. A Pearson correlation coefficient value of 0.4 was calculated for the two chemical screens. (F) Synthetic Scheme of BIBR1532. Please refer to Supplemental

Experimental Procedures for details. (G) Representative growth profiles of *$pdr1_{DBD}$-cyc8* and W303-1a cells treated with a 2-fold dilution of BIBR1532 (lanes 2-7; 0.03, 0.06, 0.12, 0.24, 0.48 and 1 μM), 2% v/v DMSO (lane 1) or untreated (lane 8; cells alone). Y-axis, optical density at 595 nm ($O.D._{595}$) with $O.D._{595}$ at 0.8 indicated; x-axis, time elapsed in minutes with 12.5 h time-point indicated.

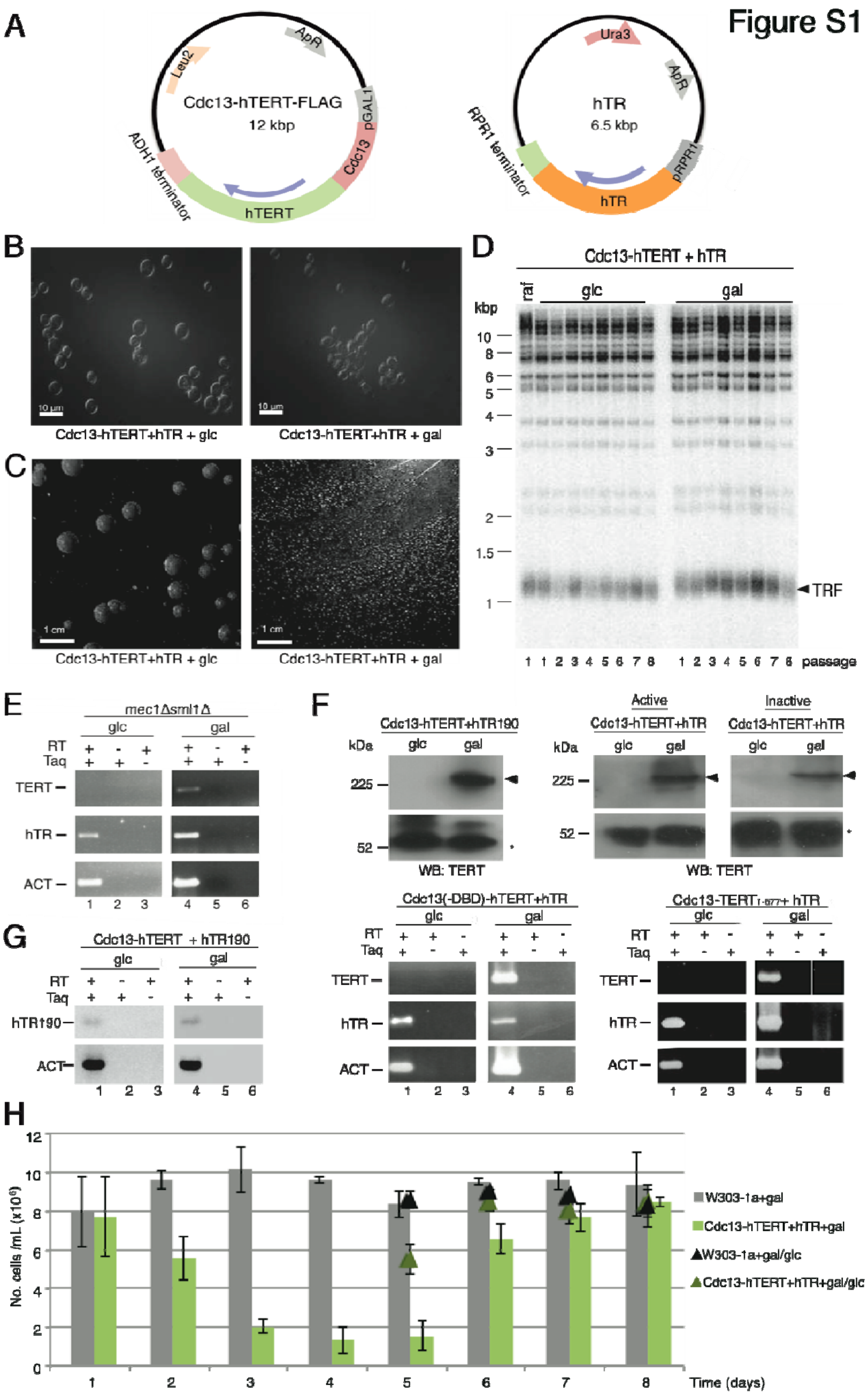
Figure S1
A
Cdc13-hTERT-FLAG
12 kbp
ApR
Leu2
pGAL1
Cdc13
ADH1 terminator
hTERT
hTR
6.5 kbp
Ura3
ApR
RPR1 terminator
pRPR1
hTR
B
Cdc13-hTERT+hTR + glc
Cdc13-hTERT+hTR + gal
C
Cdc13-hTERT+hTR + glc
Cdc13-hTERT+hTR + gal
D
Cdc13-hTERT + hTR
raf
glc
gal
kbp
TRF
passage
E
mec1Δsml1Δ
glc
gal
RT
Taq
TERT
hTR
ACT
F
Cdc13-hTERT+hTR190
kDa
WB: TERT
Active
Inactive
Cdc13-hTERT+hTR
Cdc13(-DBD)-hTERT+hTR
Cdc13-TERT1-677+ hTR
G
Cdc13-hTERT + hTR190
hTR190
ACT
H
No. cells /mL (x10⁶)
Time (days)
W303-1a+gal
Cdc13-hTERT+hTR+gal
W303-1a+gal/glc
Cdc13-hTERT+hTR+gal/glc

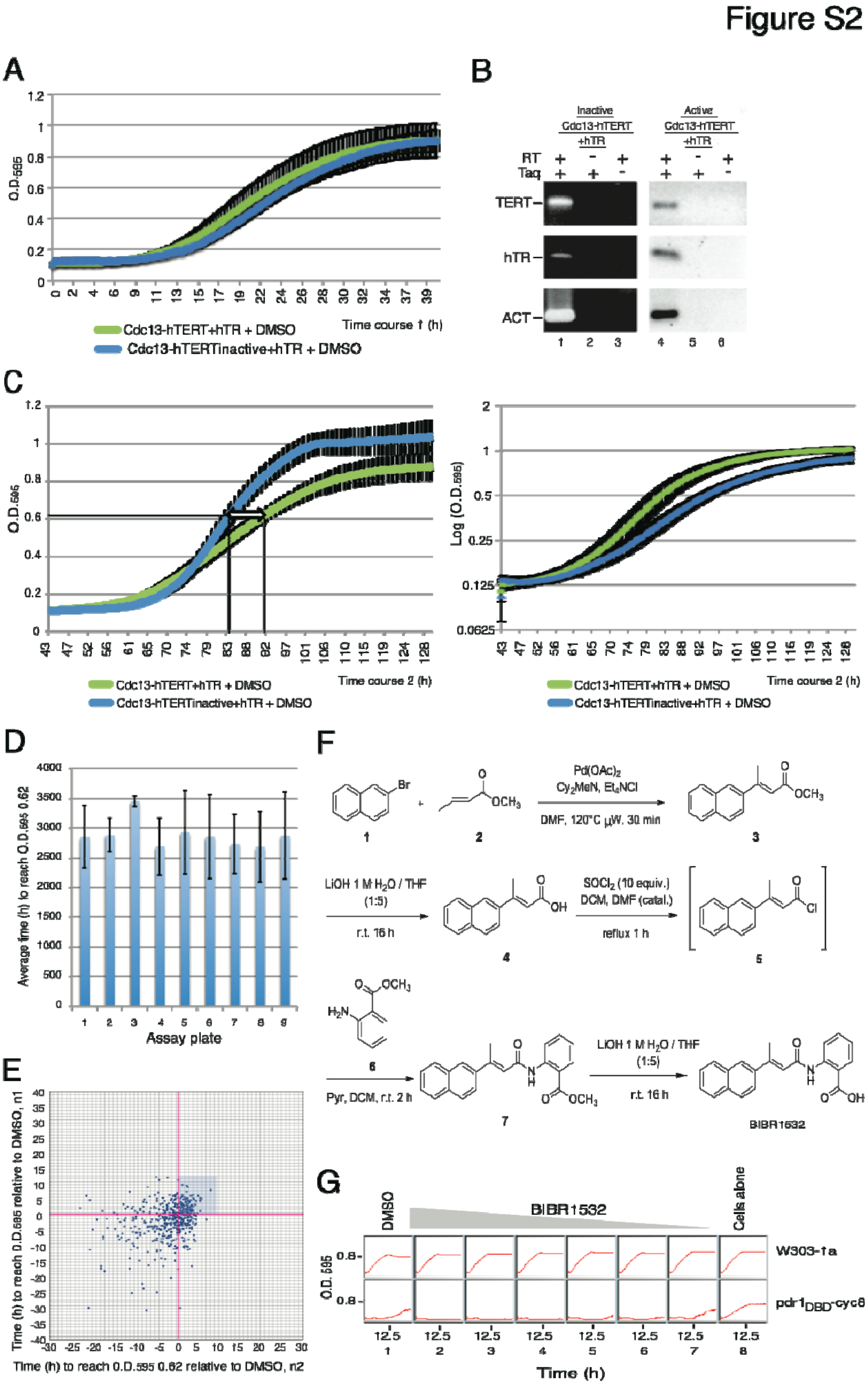
Figure S2
A
O.D.595
Time course 1 (h)
Cdc13-hTERT+hTR + DMSO
Cdc13-hTERTinactive+hTR + DMSO
B
Inactive Cdc13-hTERT +hTR
Active Cdc13-hTERT +hTR
RT
Taq
TERT
hTR
ACT
C
O.D.595
Log (O.D.595)
Time course 2 (h)
Cdc13-hTERT+hTR + DMSO
Cdc13-hTERTinactive+hTR + DMSO
D
Average time (h) to reach O.D.595 0.62
Assay plate
E
Time (h) to reach O.D.595 0.62 relative to DMSO, n1
Time (h) to reach O.D.595 0.62 relative to DMSO, n2
F
Pd(OAc)2
Cy2MeN, Et4NCl
DMF, 120°C μW, 30 min
LiOH 1 M H2O / THF (1:5)
r.t. 16 h
SOCl2 (10 equiv.)
DCM, DMF (catal.)
reflux 1 h
Pyr, DCM, r.t. 2 h
BIBR1532
G
DMSO
BIBR1532
Cells alone
O.D. 595
W303-1a
pdr1DBD-cyc8
Time (h)

Supplemental Experimental Procedures

Follow-up analysis of compounds identified in HTS

From the high-throughput analysis, 51 compounds with a neutral effect or that rescued the growth of strains expressing active human telomerase (relative to DMSO) were picked from the master HTS plates and re-analyzed (Figure 2C, and data not shown). These compounds were tested on a control strain, W303-1a expressing hTR and Cdc13-hTERT lacking the Cdc13 DBD, and 7 compounds were excluded from further analysis because they elicited a non-specific growth delay of the control strain. The remaining 44 compounds were subjected to secondary screens against the query strain expressing active human telomerase (n=2). Based on these results, twenty-eight compounds were purchased for further verification against the control and query strain, and 3 HTS hits continued to exhibit a negligible effect on growth of the control strain (-2 to + 2 h relative to DMSO) and rescued growth of the query strain by 8 h or more. Furthermore, one compound that did not rescue growth in the HTS, SEW05920, was included in follow-up analysis because it rescued growth of the query strain by 8 h when re-plated from the master HTS plate (n=2) and after the compound was re-purchased (n=4), and it was demonstrated to be a *bona fide* telomerase inhibitor *in vitro*. These four compounds are listed in Table 1.

Telomerase elongation activity *in vitro*

Telomerase activity was assessed using the Telomere Repeat Amplification Protocol (TRAPeze, Millipore) or ELISA-TRAP (TeloTAGGG Telomerase PCR ELISA-PLUS, Roche Diagnostics) following manufacturers' instructions. Compounds, where added, were incubated with telomerase for 15 minutes at room temperature prior to addition of the oligonucleotide substrate.

Terminal Restriction Fragment Analysis by Southern blot

Genomic DNA was extracted from cells as described in Philippsen 1991 (Philippsen et al., 1991), digested with XhoI (New England Biolabs) and subjected to Southern blot analysis with a yeast telomeric probe (5'-CCACACCCACACCCACAC-3') end-labelled with γ-32P-dATP as described previously (Lebel et al., 2009). The washed membrane was exposed to a phosphorimager screen overnight and scanned using the Fluoro Image Analyzer FLA-5000.

Cell inviability assays

Phloxine B (10 μg/mL) was added to selective agar plates to assess the fraction of inviable colonies based on the incorporation of the red color Phloxine B dye (Tsukada and Ohsumi, 1993). Trypan blue exclusion assay was used to assess inviable cells in liquid culture by counting the number of trypan blue-stained cells with compromised cell membrane potential (Karpova et al., 1993).

Synthesis of 2-[(*E*)-3-naphthalen-2-yl-but-2-enoylamino]-benzoic acid (BIBR1532)

BIBR1532 was synthesized following a synthetic route based on slightly modified procedures found in the literature (Barma et al., 2003; Gürtler and Buchwald, 1999) with an overall yield of 50% (Supplementary Figure 5). BIBR was > 95% pure according to elemental analysis, which showed values within the established purity limits (< 0.4% variation of the calculated values). 2-Bromo-naphthalene, **1**, (5.5 mmol, 1,114 mg), methyl crotonate, **2**, (5 mmol, 500 mg), tetraethylammonium chloride (5 mmol, 828 mg), *N*,*N*-dicyclohexylmethylamine (7.5 mmol, 1.6 mL) and palladium acetate (0.15 mmol, 33 mg) were added into an oven-dried 10-20 mL

Biotage's microwave vial with a magnetic stirrer. Subsequently, 20 mL of peptide synthesis-grade DMF was added and the mixture microwave irradiated for 30 min at 120°C. The reaction mixture was cooled down to room temperature (r.t.), diluted in ether (50 mL) and washed with distilled water (3 x 50 mL). The crude was purified by flash chromatography (EtOAc / hexanes 1:3 to 1:2, in 4 cm column) to afford methyl (*E*)-(2-naphthyl)-3-methylacrylate, **3**, as a white solid. Yield: 790 mg (71%, *E*/*Z* ratio 6:1). ^{1}H NMR and MS spectral data of compound **3** were identical to the literature values (Gürtler and Buchwald, 1999). Compound **3** (1.75 mmol, 395 mg) was dissolved in a 1:5 mixture of 1 M LiOH aqueous solution and THF (25 mL) and the reaction mixture stirred at room temperature for 16 h. The resulting mixture was then diluted in 40 mL of distilled water, acidified to pH 4 using a 1 N HCl solution and extracted with EtOAc (3 x 40 mL). The combined organic extracts were dried over sodium sulphate anhydrous and evaporated under reduced pressure to give (*E*)-(2-naphthyl)-3-methylacrylic acid, **4**, as a white powder (LRMS (ES-): *m/z* 211.1 [100, $(M-H)^-$]). Without further purification, compound **4** (1.5 mmol, 318 mg) was dissolved in a mixture of dry DCM and $SOCl_2$ (8:2, 20 mL). 2 drops of DMF were subsequently added and the mixture refluxed for 2 h. After cooling down, all volatiles were removed under vacuum and the crude acid chloride **5** re-dissolved in 20 mL of dry DCM. Methyl anthranilate, **6**, (1.5 mmol, 226 mg) and pyridine (1.6 mmol, 130 μL) were then added and the reaction stirred at room temperature for 2 h under argon atmosphere. The reaction mixture was poured into 20 mL of 0.1 N HCl, extracted with Et_2O (3 x 40 mL), and the combined organic extracts dried over sodium sulphate anhydrous and evaporated in vacuo. The crude was purified by flash chromatography (EtOAc / hexanes 1:3 to 1:2, in 3 cm column) to afford methyl 2-[(*E*)-3-naphthalen-2-yl-but-2-enoylamino]-benzoate, **7**, as a light yellow solid. Yield: 404 mg (78%, two steps). ^{1}H NMR and MS spectral data of compound **7** were identical to

published literature values(Barma et al., 2003). Compound **7** (1 mmol, 345 mg) was dissolved in a 1:5 mixture of 1 M LiOH aqueous solution and THF (20 mL) and the reaction mixture stirred at room temperature for 16 h. The resulting mixture was then diluted in 30 mL of distilled water, acidified to pH 4 using a 1 N HCl solution and extracted with EtOAc (3 x 40 mL). The combined organic extracts were dried over sodium sulphate anhydrous, evaporated under reduced pressure and the resulting crude purified by flash chromatography (EtOAc / hexanes 1:1, in 3 cm column) to afford 2-[(*E*)-3-naphthalen-2-yl-but-2-enoylamino]-benzoic acid, BIBR1532, as a white solid. Yield: 298 mg (90%). ^{1}H NMR ($CDCl_3$, 250 MHz) δ 11.10 (s, 1H), 8.83 (dd, 1H, *J* (Hz) = 0.9, 8.5), 8.06 (dd, 1H, *J* (Hz) = 1.5, 8.0), 7.91–7.74 (m, 4H), 7.60–7.38 (m, 4H), 7.17 (t, 1H, *J* (Hz) = 8.5), 6.30 (d, 1H, *J* (Hz) = 1.2), 2.69 (d, 3H, *J* (Hz) = 1.2). LRMS (ES-): *m/z* 330.0 [100, $(M-H)^-$], 661.1 [15, $(2M-H)^-$]. BIBR1532 spectroscopic data matched with published literature values(Barma et al., 2003). Elemental Analysis of BIBR1532 ($C_{21}H_{17}NO_3$): expected, C 76.12, H 5.17, N 4.23, O 14.49; found, C 76.05, H 5.33, N 4.01, O 14.65.

The effect of BIBR1532 on *pdr1$_{DBD}$-cyc8* by growth assay

Pdr1$_{DBD}$-cyc8 and W303-1a cells were grown in synthetic complete media with 2% v/v glucose until an O.D.$_{600}$ 0.3. Cells were transferred into a 96 well clear flat-bottomed microplate (Corning Costar) containing fresh synthetic complete media with 2% v/v glucose and BIBR1532 compound at a final concentration of 0.03, 0.06, 0.12, 0.24, 0.46 and 1 μM. Cells alone and with 2% v/v DMSO were added as controls in wells. Plate was sealed and read overnight by a Sunrise plate reader (Tecan) at absorbance 595 nm, maintained at 30°C and shaking at 564 r.p.m.

Supplemental Table

Plasmid (marker/vector)
phTR (*URA3*/pIIIEx426)
phTR190 (*URA3*/pIIIEx426)
pGAL1-Cdc13-hTERT-FLAG (*LEU2*)
pGAL1-Cdc13-TERT(D868A)-FLAG (*LEU2*)
pGAL1-Cdc13(-DBD)-hTERT (*LEU2*)
Empty *GAL1* promoter (*TRP5*)(Longtine et al., 1998)
pCR3-FLAG-hTERT-FLAG(Beattie et al., 1998)
pCR3-hTERT-FLAG 5-1(Harrington et al., 1997)
pVL1091(Evans and Lundblad, 1999)
pUC19-hTR(Beattie et al., 2000)

Table S1. Plasmids employed in this study. Plasmids were prepared by cloning in *E. coli* DH5α or TOP10F’ strains and isolated using QIAprep Spin Miniprep Kit (QIAGEN® Chatsworth, CA). Plasmids carrying genes of interest were confirmed by sequencing.

Supplemental References

Barma, D.K., Elayadi, A., Falck, J.R., and Corey, D.R. (2003). Inhibition of telomerase by BIBR 1532 and related analogues. Bioorg. Med. Chem. Lett. 13, 1333-1336.

Gürtler, C., and Buchwald, S.L. (1999). A Phosphane-Free Catalyst System for the Heck Arylation of Disubstituted Alkenes: Application to the Synthesis of Trisubstituted Olefins. Chem. Eur. J. 5, 3107-3112.

Hayflick, L. (1973). Aging human cells. Triangle 12, 141-147.

Karpova, T.S., Lepetit, M.M., and Cooper, J.A. (1993). Mutations that enhance the cap2 null mutant phenotype in *Saccharomyces cerevisiae* affect the actin cytoskeleton, morphogenesis and pattern of growth. Genetics 135, 693-709.

Lebel, C., Rosonina, E., Sealey, D.C., Pryde, F., Lydall, D., Maringele, L., and Harrington, L.A. (2009). Telomere maintenance and survival in *Saccharomyces cerevisiae* in the absence of telomerase and RAD52. Genetics 182, 671-684.

Longtine, M.S., McKenzie, A., 3rd, Demarini, D.J., Shah, N.G., Wach, A., Brachat, A., Philippsen, P., and Pringle, J.R. (1998). Additional modules for versatile and economical PCR-based gene deletion and modification in *Saccharomyces cerevisiae*. Yeast 14, 953-961.

Philippsen, P., Stotz, A., and Scherf, C. (1991). DNA of *Saccharomyces cerevisiae*. Methods Enzymol. 194, 169-182.

Tsukada, M., and Ohsumi, Y. (1993). Isolation and characterization of autophagy-defective mutants of *Saccharomyces cerevisiae.* FEBS Lett. 333, 169-174.
